# Women’s experiences of participating in a randomised trial comparing alternative policies for timing of cord clamping at very preterm birth: a questionnaire study

**DOI:** 10.1186/s13063-019-3325-4

**Published:** 2019-04-16

**Authors:** Lucy Bradshaw, Alexandra Sawyer, Eleanor Mitchell, Lindsay Armstrong-Buisseret, Susan Ayers, Lelia Duley

**Affiliations:** 1 Nottingham Clinical Trials Unit, University of Nottingham, Nottingham, NG7 2UH UK; 2 School of Health Sciences, University of Brighton, Falmer, BN1 9PH UK; 3 Centre for Maternal and Child Health Research, School of Health Sciences, City University London, London, EC1V 0HB UK

**Keywords:** Preterm birth, Umbilical cord-clamping, Neonatal care with cord intact, Experience, Clinical trials

## Abstract

**Background:**

The Cord Pilot Trial compared two alternative policies for cord-clamping at very preterm birth at eight UK tertiary maternity units: clamping after at least 2 min and immediate neonatal care with cord intact, or clamping within 20 s and neonatal care after clamping. This paper reports views and experiences of the women who participated in the trial (261 randomised), based on data from two self-completed questionnaires.

**Methods:**

Women were given or posted the first questionnaire between 4 and 8 weeks after birth, and posted a second similar questionnaire at 1 year. Both questionnaires included three questions about experiences of participating in the trial: (1) If time suddenly went backwards and you had to do it all over again, would you agree to participate in the Cord Pilot Trial?; (2) Please tell us if there was anything about the Cord Pilot Trial that you think could have been done better; and (3) Please tell us if there was anything about the Cord Pilot Trial, or your experiences of joining the trial, that you think were particularly good.

**Results:**

One hundred and eighty-six women completed the first questionnaire and 133 completed the second. At both time points, 90% responded ‘probably‘ or ‘definitely‘ to participating in the trial again. More women randomised to deferred clamping responded ‘definitely yes‘ than those allocated immediate clamping (78% versus 67% first questionnaire). Women were positive about the level of information and explanations, the friendly and caring staff, and the benefits for their baby and others as a result of participating in the trial. Suggestions for how the trial could be done better included being approached earlier, better staff communication about the trial, more information overall, and better timing of follow-up.

**Conclusions:**

Women were largely positive about participating in the trial. Nevertheless, they had suggestions for how the study could have been improved. These suggestions have implications for the design of future trials.

**Trial registration:**

ISRCTN21456601. Registered on 28 February 2013.

**Electronic supplementary material:**

The online version of this article (10.1186/s13063-019-3325-4) contains supplementary material, which is available to authorized users.

## Background

The Cord Pilot Trial was a randomised comparison of alternative policies for the timing of cord-clamping at very preterm birth [1–3] at eight UK tertiary maternity units. The trial included two novel elements which may have influenced women’s experiences of participation. First, to allow deferred cord-clamping without delaying neonatal care for babies requiring resuscitation at birth, newborn life support at birth was provided with cord intact and thus also allowed women to share the first moments of their baby’s life [4, 5]. Second, so that high-risk women and babies could be offered the opportunity to participate, we developed a two-stage consent pathway (with oral assent before the birth and written consent after the birth) for use when birth was imminent [6, 7]; this pathway was used for almost one third of recruitment [3].

Randomised trials are the ‘gold standard’ for evaluation of healthcare interventions. Understanding the experiences of women recruited to trials during pregnancy is especially important as they are also making a decision on behalf of their unborn child [8]. Conducting trials in stressful circumstances, such as when birth is likely to be very preterm, is challenging. This is particularly so when time for offering participation is limited; for example, when birth is imminent [9]. Better understanding of women’s experiences may help improve the design of trials and their relevance to the participants, thereby minimising potential barriers to participation, improving the experience of participating and improving recruitment and retention.

This paper reports the views and experiences of women about participating in the Cord Pilot Trial, based on self-completed questionnaires up to 1 year after the birth.

## Methods

The Cord Pilot Trial compared cord-clamping after at least 2 min and providing immediate neonatal care, if needed, with cord intact (deferred clamping) with clamping within 20 s and neonatal care after clamping (immediate clamping) at very preterm birth (< 32 weeks gestation) [1, 2]. Initially, the objective was to assess the feasibility of conducting a large, multicentre, UK trial and, as feasibility was demonstrated, recruitment continued whilst funding for the full trial was sought. The trial closed when the funding application was unsuccessful [10]. Throughout the planning and conduct of the trial, we worked with the National Childbirth Trust and Bliss (a UK-based charity for babies born prematurely or sick) for a strong parent perspective.

Posters about the study and summary information sheets were available in antenatal clinics and on antenatal wards. Women at risk of very preterm birth were invited to participate; if they accepted they gave written consent. Eligibility and willingness to participate were checked before randomisation, which was during labour or at caesarean section. If birth was imminent and the attending clinician felt it appropriate, women were offered a brief description of the trial and offered participation (oral assent). Those who gave oral assent were then randomised. After the birth, these women had an opportunity to discuss the study in more detail, and were invited to give written consent for participation in follow-up. This two-stage consent pathway was developed in discussion with the National Childbirth Trust and Bliss, to be used only when there was insufficient time for the usual consent process, giving these women the opportunity to participate [1].

For follow-up, women were asked to complete two similar questionnaires, the first between 4 and 8 weeks after giving birth, the second at 1 year. We initially planned that the first questionnaire would be posted to the woman’s home 6 weeks after the birth, but this often coincided with discharge of the baby which was not a good time for the women to receive it. Therefore, we changed this and if the baby was still in hospital at age 4 weeks, the research midwife/nurse gave the questionnaire to the woman when she was visiting. If the baby was not in hospital at 4 weeks, we posted it to her 8 weeks after the birth. If the baby died or was stillborn, covering letters and the questionnaires were adapted appropriately. Before sending the questionnaire at 1 year, we checked the baby’s status with the recruiting site, or with the general practitioner if the family had not been seen recently at the site. We then posted the questionnaire, along with a birthday card for the child (if appropriate). A stamped addressed envelope was provided to return completed questionnaires. If there was no response, we sent a postal reminder after 2 weeks. If there was still no response after another 2 weeks, we telephoned the woman and offered the opportunity to complete the questionnaire over the telephone. If no telephone number was available, we sent a second postal reminder.

Both questionnaires included three questions about the women’s experience of participating in the trial, which had been previously used for a trial involving women with pre-eclampsia [11]:

1. If time suddenly went backwards and you had to do it all over again, would you agree to participate in the Cord Pilot Trial? (response options: ‘definitely yes’, ‘probably yes’, ‘not sure’, ‘probably no’, and ‘definitely no’. Free text to explain response)
2. Please tell us if there was anything about the Cord Pilot Trial that you think could have been done better (free-text response)
3. Please tell us if there was anything about the Cord Pilot Trial, or your experience of joining the trial, that you think was particularly good (free-text response)

Responses to other questions about symptoms of anxiety or depression, satisfaction with care at birth, and breastfeeding/expressing are reported elsewhere [12].

### Data analysis

For the analysis of outcomes to discharge, women who gave birth after 35^+ 6^ weeks gestation were excluded as outcomes for these babies are different from those born very preterm [3]. These women are, therefore, also excluded here. The number of questionnaires returned was described, along with the proportion of women completing each of the three questions about participation. We pre-specified factors that might influence women’s experiences [1], with primary variables being death of the baby and the allocated group. Secondary variables were maternal age at recruitment, gestation at recruitment less than 30 weeks, whether the two-stage consent pathway was used, severe postpartum haemorrhage (blood loss > 1000 ml), possible depression (Hospital Anxiety and Depression Scale depression score ≥ 8 [13]), length of stay in neonatal intensive care unit longer than 6 weeks, and need for a reminder to complete the questionnaire. Responses about whether women would participate again were tabulated overall and according to these pre-specified variables for the two questionnaires.

We coded the free-text responses and grouped responses within themes using inductive content analysis, which involved the following steps: (1) all responses were read twice for each question; (2) line-by-line analysis summarising the content of each statement; (3) a coding scheme based on the content of women's responses was created; and (4) responses were then coded using the coding scheme. For Question 1, respondents were asked to explain why they had chosen a particular response category; therefore, these free-text responses were grouped according to the fixed-response category that they were linked to. The number with comments within each theme are presented according to allocated group and consent pathway (usual one-stage or two-stage). We collated and analysed responses using Microsoft Excel which is a useful tool for grouping open-ended responses into categories that can be summarised qualitatively and quantitatively [14].

## Results

**Fig. 1:**
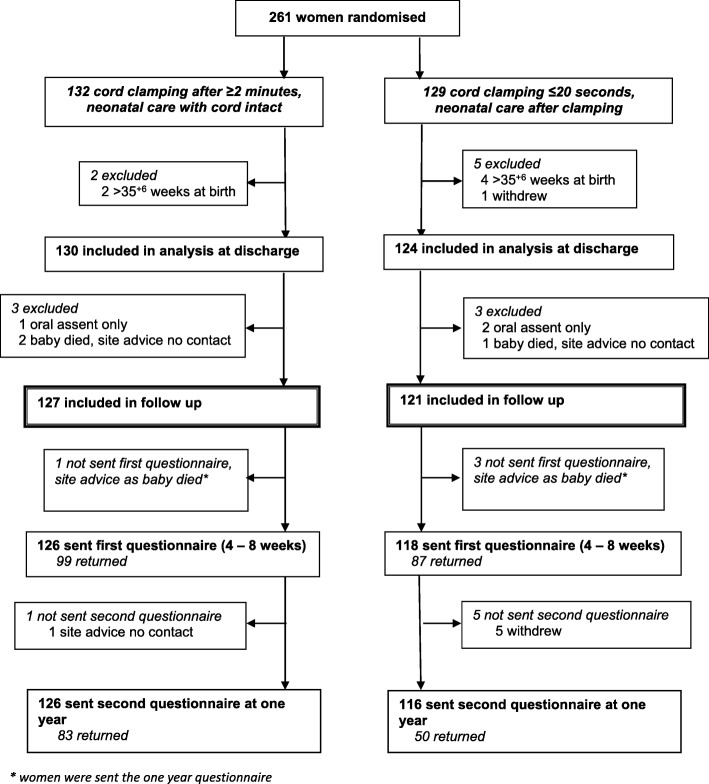
Consolidated Standards of Reporting Trials (CONSORT) flow for the follow-up of women to 1 year with questionnaires

Overall, 261 women were randomised between March 2013 and February 2015, six of whom were excluded as they gave birth after 35^+ 6^ weeks and one withdrew the use of their data, leaving 254 women for the analysis of outcome at hospital discharge (Fig. 1) [3]. A further six women were excluded from follow-up after discharge: three for whom we had oral assent only and so consent for follow-up was not available, and three whose baby died before discharge and the site advised us not to contact them. Four women were sent the 1-year questionnaire only, as their baby died and the site advised us not to send the first questionnaire.

Of 244 women sent the first questionnaire, four did not complete it and asked not to be contacted again, another completed it but asked for no further follow-up. Overall, 186 of the first questionnaires (76%) were returned (79% deferred clamping, 74% immediate clamping) (Fig. 1). For one woman the site advised us not to send the second questionnaire due to safeguarding issues. Two hundred and forty-two women were sent the second questionnaire at 1 year, and 133 (55%) responded (66% deferred clamping, 43% immediate clamping). Although the questionnaires asked about the women, a few were completed by someone else on their behalf (six of the first questionnaires and five of the second). For six of the first questionnaires and three of the second, it was not stated who completed the questionnaire.

For both questionnaires, characteristics at trial entry for the women who responded were similar between the allocated groups (Table 1). A higher proportion of women allocated deferred clamping responded than those allocated immediate clamping, and this was more marked for the second questionnaire at 1 year. Factors in response were similar in both groups and have been described in detail elsewhere: response was higher for women aged 30 years or older at the time that they gave consent, and for those recruited during their first pregnancy lasting 20 weeks or more [12]. Response for the first questionnaire was similar, although slightly lower, for women recruited using the two-stage consent pathway (69%) and the usual one-stage consent pathway (78%). Response for the second questionnaire was very similar for the two consent pathways (56% for usual one-stage and 53% for two-stage).

**Table 1:** For women who returned questionnaires: baseline characteristics, baby’s length of stay and baby status

	First (at 4–8 weeks)		Second (at 1 year)	
	Clamp ≥ 2 min + neonatal care with cord intact	Clamp ≤ 20 s + neonatal care after clamping	Clamp ≥ 2 min + neonatal care with cord intact	Clamp ≤ 20 s + neonatal care after clamping
	*n* = 99 (%)	*n* = 87 (%)	*n* = 83 (%)	*n* = 50 (%)
Age (years)				
< 20	2 (2%)	7 (8%)	1 (1%)	1 (2%)
20–24	14 (14%)	14 (16%)	11 (13%)	6 (12%)
25–29	19 (19%)	21 (24%)	13 (16%)	12 (24%)
30–34	41 (41%)	29 (33%)	38 (46%)	16 (32%)
35–39	18 (18%)	10 (11%)	15 (18%)	9 (18%)
≥ 40	5 (5%)	6 (7%)	5 (6%)	6 (12%)
Consent pathway				
usual one-stage	73 (74%)	68 (78%)	60 (72%)	39 (78%)
two-stage	26 (26%)	19 (22%)	23 (28%)	11 (22%)
Gestation at birth (weeks)				
< 26	15 (15%)	8 (9%)	12 (14%)	4 (8%)
26^+ 0^–27^+ 6^	22 (22%)	16 (18%)	16 (19%)	8 (16%)
28^+ 0^–29^+ 6^	27 (27%)	34 (39%)	22 (27%)	20 (40%)
30^+ 0^ - 31^+ 6^	34 (34%)	28 (32%)	31 (37%)	16 (32%)
≥ 32	1 (1%)	1 (1%)	2 (2%)	2 (4%)
Blood loss at birth (ml)				
0–499	49 (49%)	44 (51%)	41 (49%)	23 (46%)
500–999	41 (41%)	33 (38%)	34 (41%)	21 (42%)
≥ 1000	9 (9%)	10 (11%)	8 (10%)	6 (12%)
For baby:				
length of hospital stay^1^ (weeks)				
≤ 6	27 (27%)	24 (28%)	25 (30%)	16 (32%)
> 6	70 (71%)	61 (70%)	56 (67%)	33 (66%)
died before discharge	2 (2%)	2 (2%)	2 (2%)	1 (2%)
status when questionnaire completed^2^				
alive	98 (99%)	83 (95%)	81 (98%)	48 (96%)
dead	1 (1%)	4 (5%)	2 (2%)	2 (4%)

1 – For twin births, category is based on longest stay. If one twin died, category is based on stay of surviving twin, this occurred for two families completing the postnatal questionnaire and one family completing the 1-year questionnaire

2 – For twin births, if one twin died status is reported according to the deceased twin. Note, for one participant the first questionnaire was completed when the baby was still in hospital who later died. Number of babies who died includes stillbirths

### ‘If time suddenly went backwards, and you had to do it all over again, would you agree to participate in the Cord Pilot Trial?’

Overall, responses to this question were positive, with more than 90% of respondents saying ‘probably yes’ or ‘definitely yes’ on both questionnaires (Table 2). Responses were similar for women recruited during the feasibility phase, and during the extended recruitment phase. However, for both questionnaires more women allocated deferred clamping responded ‘definitely yes’ than those allocated immediate clamping (78% versus 67% on the first questionnaire, 84% versus 69% on the second) (Table 2). Of 113 women who completed this question on both questionnaires, 81 (71%) responded ‘definitely yes’ on both. There were no clear differences according to any pre-specified factors, including the consent pathway (Table 2).

**Table 2:** Response to Question 1, overall and according to factors that might influence experience

	‘If time suddenly went backwards, and you had to do it all over again, would you agree to participate in the Cord Pilot Trial?’									
	First (at 4–8 weeks)					Second (at 1 year)				
	Definitely no	Probably no	Not sure	Probably yes	Definitely yes	Definitely no	Probably no	Not sure	Probably yes	Definitely yes
Total	2 (1%)	1 (1%)	9 (5%)	36 (20%)	131 (73%)	1 (1%)	2 (2%)	6 (5%)	19 (15%)	103 (79%)
Clamping after ≥ 2 min + neonatal care with cord intact	1 (1%)	–	3 (3%)	17 (18%)	75 (78%)	–	1 (1%)	1 (1%)	11 (13%)	69 (84%)
Clamping ≤ 20 s + neonatal care after clamping	1 (1%)	1 (1%)	6 (7%)	19 (23%)	56 (67%)	1 (2%)	1 (2%)	5 (10%)	8 (16%)	34 (69%)
Gestation at birth *(*weeks) < 26	–	1 (4%)	1 (4%)	3 (13%)	18 (78%)	–	–	1 (7%)	3 (20%)	11 (73%)
26^+ 0^–27^+ 6^	–	–	2 (5%)	8 (22%)	27 (73%)	–	1 (4%)	–	2 (9%)	20 (87%)
28^+ 0^–29^+ 6^	1 (2%)	–	2 (3%)	17 (29%)	39 (66%)	1 (2%)	–	1 (2%)	9 (21%)	31 (74%)
30^+ 0^ –31^+ 6^	1 (2%)	–	4 (7%)	8 (14%)	45 (78%)	–	1 (2%)	3 (6%)	5 (11%)	38 (81%)
≥ 32	–	–	–	–	2 (100%)	–	–	1 (25%)	–	3 (75%)
Consent pathway										
usual one-stage	2 (1%)	1 (1%)	6 (4%)	28 (21%)	99 (73%)	1 (1%)	2 (2%)	6 (6%)	8 (8%)	81 (83%)
two-stage	–	–	3 (7%)	8 (19%)	32 (74%)	–	–	–	11 (33%)	22 (67%)
Age at trial entry (years) < 20	–	–	1 (11%)	3 (33%)	5 (56%)	–	–	1 (50%)	–	1 (50%)
20–24	–	–	1 (4%)	5 (19%)	20 (77%)	–	–	1 (6%)	4 (24%)	12 (71%)
25–29	–	1 (3%)	2 (5%)	4 (11%)	31 (82%)	–	1 (4%)	2 (8%)	1 (4%)	21 (84%)
30–34	2 (3%)	–	4 (6%)	15 (22%)	47 (69%)	–	1 (2%)	1 (2%)	8 (15%)	43 (81%)
35–39	–	–	–	7 (26%)	20 (74%)	–	–	–	5 (22%)	18 (78%)
≥ 40	–	–	1 (9%)	2 (18%)	8 (73%)	1 (9%)	–	1 (9%)	1 (9%)	8 (73%)
Blood loss at birth (ml) 0–499	1 (1%)	1 (1%)	4 (4%)	18 (20%)	65 (73%)	–	–	5 (8%)	10 (16%)	48 (76%)
500–999	–	–	4 (6%)	16 (23%)	51 (72%)	–	2 (4%)	1 (2%)	7 (13%)	44 (81%)
≥ 1000	1 (5%)	–	1 (5%)	2 (11%)	15 (79%)	1 (7%)	–	–	2 (14%)	11 (79%)
For baby, length of stay > 6 weeks	1 (1%)	1 (1%)	4 (3%)	27 (21%)	95 (74%)	1 (1%)	1 (1%)	3 (3%)	13 (15%)	69 (79%)
Hospital Anxiety and Depression Scale depression score ≥ 8	1 (2%)	–	6 (12%)	9 (17%)	36 (69%)	–	–	–	5 (20%)	20 (80%)
Baby died before questionnaire completed (including stillbirths)	–	–	1 (25%)	1 (25%)	2 (50%)	–	–	–	1 (25%)	3 (75%)
Questionnaire completed after reminder	2 (2%)	–	3 (4%)	17 (21%)	59 (73%)	1 (1%)	1 (1%)	6 (7%)	14 (17%)	60 (73%)
Recruited period										
First year (feasibility phase)	1 (1%)	–	4 (5%)	19 (22%)	62 (72%)	–	1 (1%)	5 (7%)	10 (14%)	54 (77%)
Second year	1 (1%)	1 (1%)	5 (5%)	17 (18%)	69 (74%)	1 (2%)	1 (2%)	1 (2%)	9 (15%)	49 (80%)

Note:

▪ 7 of the 186 participants returning the first questionnaire did not complete this question including one of the participants whose baby died (3 in the clamp cord after at least 2 min group and 4 in the clamp cord within 20 s group)

▪ 2 of the 132 participants returning the second questionnaire at 1 year did not complete this question (1 in each group)

Of those who provided free-text explaining their responses, on the first questionnaires the main reasons for responding ‘definitely yes’ were that research is important (*n* = 34), benefits to the baby (*n* = 26), benefits to others (*n* = 26), and no risk to the baby (*n* = 22) (see Additional file 1: Table S1). Another relatively common reason was that women thought the trial was important and/or were positive about deferred cord-clamping (*n* = 12). For example, some women had already planned for deferred cord-clamping if their pregnancy went to term. Some women reported that being in the trial was a positive experience (*n* = 10) and others mentioned the comfort in seeing their baby being cared for beside them (*n* = 3). Reasons for responses of ‘probably yes’ were similar. One respondent selected ‘probably no’, and said this was because she thought the trial had no impact (positive or negative). Two women responded ‘definitely no’; for one this was because of disappointment with the allocated group, the other had a strong preference for one intervention. One of these respondents also felt hounded by trial staff.

For the second questionnaire at 1 year, women’s reasons for responding ‘definitely yes’ were similar: benefit to others (*n* = 35), the importance of research (*n* = 23), benefit to the baby (*n* = 22), and no risk to the baby (*n* = 10). They also said that the trial not having any impact on them and/or the birth experience was a factor. The two women who selected ‘probably no’ explained that this was because once their baby was born the trial felt less like a priority. No reason was given for the single response of ‘definitely no’ (see Additional file 1: Table S2).

The first questionnaire was completed by five women whose baby had died and the second questionnaire at 1 year by four women. Women whose baby died responded either ‘not sure’, ‘probably yes’ or ‘definitely yes’ to whether they would participate in the trial again (Table 2). The small number of responses from women whose baby died did not allow qualitative differences in responses to the three open-ended questions between bereaved and non-bereaved parents to be explored.

### ‘Please tell us if there was anything about the Cord Pilot Trial that you think could have been done better’

Of women completing the first questionnaire, two thirds (125/186, 67%) responded to this question (67 deferred clamping, 58 immediate clamping); of whom three quarters (93/125, 74%) said that they did not think that anything could have been done better. Of the 32 who suggested that things that could have been done better, the main themes were: to approach women earlier or at a different time to join the trial (*n* = 9); to explain afterwards which intervention they received (*n* = 4); better staff communication about the trial (*n* = 3); and provide more information (*n* = 3) (Table 3). Many of the women who gave suggestions for how the trial could have been done better responded either *‘definitely yes’* (*n* = 23) or ‘*probably yes’* (*n* = 4) to Question 1.

**Table 3:** For the first questionnaire, summary of responses about what could have been done better

	Consent pathway		Allocated group		Sample of comments
	Usual one-stage *n* = 97	Two-stage *n* = 28	Clamping ≥ 2 min *n* = 67	Clamping ≤ 20 s *n* = 58	
Nothing could have been done better	67	26	51	42	‘There isn't anything that could have been done better’
Approach earlier/different time	4	5	3	6	‘I think you could approach patients in earlier stages of labour. I was approached at the point when my contractions were quite strong and frequent and it was quite hard for me to concentrate on the information about the trial’ ‘The point when we were approached about it was a bit of a stressful moment, it probably would have been better another time but it is hard to know when!’
Explain afterwards which intervention they received	4	–	3	1	‘During my section and after the doctors and nurse were at odds as to what they needed to do. That worried me at the time, but everything happened so quickly, I lost track of what happened, and it was never explained as to the outcome, which group he was drawn in’ ‘If I am honest I haven't been told anything about my Cord Trial so I don't know how long they waited? It would be interesting to know’
Better staff communication about the trial	3	–	2	1	‘It didn't seem to be handed to everyone that I was in the Cord Trial. I had to keep telling people I was in the trial. I wanted delayed clamping’ ‘Knowing if you were in the trial before you had the baby. Staff didn’t know when he came out if they could cut the cord or not, as there was a panic to find the envelope’
More information	2	1	2	1	‘Maybe explain that if the cord was short, as it was in my case, the baby would be kept on the bed and there was risk her temperature could drop as it did with (baby’s name)’ ‘Perhaps you could make some literature available to mothers who feel they'd like to read it, about delayed cord-clamping. If the mother is anxious at this point, information given verbally only can cover her head?’
No randomisation	1	1	2	–	‘Been able to choose which side you were on, however understand by it was a pick of a hat. So not a problem’
Other suggestions	2	1	1	2	‘To be prepared for short cords especially in case of early babies’ ‘To be told at the birth how long it was going to be for, not afterwards’ ‘Arrange times to go back and see a patient—don't turn up to the bed unannounced! To be honest I was under so much stress with severe pre-eclampsia for a second time and this trial just gave me more stress on top and then it turned out not to even be worth it because we didn't get the right envelope!’

For the second questionnaire at 1 year, a similar proportion responded to this question (85/133, 64%) (51 deferred clamping, 34 immediate clamping); of whom a similar proportion (61/85, 72%) said that they did not think anything could have been done better. Three women found it difficult to suggest what could have been done better: *‘I don't have anything similar to compare Cord Trial with’*. Of the 24 who made suggestions about what could be done better, these were similar to those on the first questionnaire but with the addition of a few comments about the follow-up: to approach women earlier or at a different time to join the trial (*n* = 6); to provide more updates and information about the trial (*n* = 5); better timing of the first questionnaire follow-up (*n* = 3); and decide earlier about which arm of the trial a woman was allocated to (*n* = 2) (Additional file 2). Many of the women who gave suggestions for how the trial could have been done better responded either *‘definitely yes’* (*n* = 16) or *‘probably yes’* (*n* = 6) to Question 1.

### ‘Please tell us if there was anything about the Cord Pilot Trial, or your experience of joining the trial that you think was particularly good’

Again, two thirds of respondents to the first questionnaire answered this question (122/186, 66%) (64 deferred clamping, 58 immediate clamping). Women valued detailed information and clear explanations, which they felt helped them understand the trial (*n* = 47). Many women commented that they found the staff caring and friendly (*n* = 21) using terms such as ‘friendliness’, ‘supportive’, and ‘reassuring’. A few also commented that they felt no pressure from staff to participate in the trial (*n* = 6). Women thought there were benefits to participating in the trial; they thought that the health of their baby was better as a result of taking part (these comments predominantly relate to the deferred clamping arm) (*n* = 15), and they that thought the trial would help other babies and families (*n* = 15) (Table 4).

**Table 4:** For the first questionnaire, summary of responses about what was good about the trial

	Consent pathway		Allocated group		Sample of comments
	Usual one-stage *n* = 94	Two-stage *n* = 28	Clamping ≥ 2 min *n* = 64	Clamping ≤ 20 s *n* = 58	
Good information and explanation	36	11	28	19	‘Everything was explained at length so I personally knew exactly what was happening and how things went after the cord was clamped’ ‘The way the information was given by friendly inspiring staff. The level of detail and thorough research into me as an individual regarding me being an eligible and safe candidate’ ‘The information that was given was explained exceptionally well’
Caring and friendly staff	16	5	12	9	‘The staff who explained the project were very friendly and took an interest in our family whilst we were waiting for our baby to be born’ ‘The friendliness of the staff involved’ ‘(name of member of staff) who has been lovely and supportive’ ‘The reassurance given that mine and my son's health and well-being were the most important thing’
Benefit to baby	10	5	12	3	‘I truly feel that keeping my baby attached to me made a huge difference to his health. He did not require too much help directly after birth and has so far done really well’ ‘I felt that the trial helped my daughter get stronger everyday by allowing her the extra 30% of blood. I would definitely participate again’ ‘My experience of joining the Cord Pilot Trial was an adventure because it could of helped my son and it did’
Benefit to others	12	3	9	6	‘Trials like this are great and are for the health and well-being of babies. It is nice to have taken part in trials that will better the care for babies’ ‘The thought that it could be helping other babies and their families’ ‘Being part of a trial that could help future premature babies was comforting at an emotional time’
No pressure from staff	4	2	4	2	‘I didn't feel pressured to participate’ ‘I had the time to decide if I wanted to participate’
See baby for longer	4	–	3	1	‘At the birth, seeing it in front of you, you understand why it is so important for mother and baby to be close together—bond not broken at birth’ ‘In addition to my previous response the cord trial allowed us to observe our baby straight after birth and be reassured he was able to cope well outside the womb’
Other	9	4	9	4	‘The good thing is I had a chance to join the trial and I got the knowledge from that trial. If I didn’t join I was not going to know anything about it’ ‘Joining the trial was easy. It didn't take a lot of time and I didn't even feel like we was part of trial it felt very natural’ ‘Being able to get a dvd of the baby’s first moments of life’

For the second questionnaire at 1 year, 79/133 (59%) respondents provided comments (53 deferred clamping, 26 immediate clamping). Again, the details provided were broadly similar to those on the first questionnaire. Women appreciated detailed information and clear explanations (*n* = 22), and commented on the caring and friendly staff (*n* = 20). They thought that there were benefits to being involved, for their baby (*n* = 5) and for others (*n* = 5). Women also wrote positively about the personable nature of the trial (*n* = 5), such as receiving a first birthday card for their baby. Some women felt that they had learnt new and interesting things from participating in the trial (*n* = 4) (Additional file 2).

## Discussion

Results of this questionnaire study provide insight into women’s experiences of participating in the Cord Pilot Trial. The response rate was higher for the first questionnaire, than for the second at 1 year; and at 1 year the response was much higher for those allocated deferred clamping (66% and 43%, respectively). Responses to the questions were similar at the two times frames however. Overall, women were positive about the trial and their participation, and only a few said that they would not participate again. There were no clear differences in response about participating again according to any pre-specified factors, including the consent pathway, except that a greater proportion of women allocated deferred clamping indicated that they would definitely participate again than those allocated immediate clamping. The main reasons that women gave for their positive response were altruistic (benefits to others, importance of research), benefits to the baby, and no risk to the baby. These are in line with what has been reported for other studies recruiting women into trials during pregnancy [15, 16]. Things women said they liked about the trial included detailed information and clear explanations of the study, and the caring and friendly staff. A greater proportion of women allocated deferred clamping made comments indicating that they thought that the health of their baby was better as a result of joining the trial.

For this trial, women were approached and invited to participate at a difficult time as they already knew their baby was likely to be born too early; and for some, birth was imminent. Therefore, it is unsurprising that some women said that they would prefer this approach to have been earlier and that they would have liked more information, albeit recognising that it can be hard to know when. This comment was made by women recruited using both the usual consent pathway and the two-stage pathway. Other studies have also reported that women would like information earlier. For example, one study exploring women’s experiences of an intrapartum trial in an emergency setting found that whilst women recognised that information provided during pregnancy may not be personally salient they thought that women should be given the choice whether or not to engage with the material [17]. However, in comparison, staff were concerned that giving information to all women would be an ineffective use of time and resources, and potentially distressing to many women to whom it would not be relevant [17]. Furthermore, on the second questionnaire at 1 year, some women wrote that the first questionnaire could have been timed better when it was less hectic, and that they would have liked to have received more updates about the progress and results of the trial. In a qualitative study exploring parents’ reactions to trial results, parents said that feedback was important to them because it provided further information and clarity, helped them to remember an emotional time, and acknowledged their important contribution to medical research [18]. Therefore, for a trial such as the Cord Pilot Trial where the follow-up continues long after the intervention has been completed, keeping participants up to date on trial progress and when they might expect to know the results of the trial, is particularly important. Consistent with previous research [19, 20], responses from several women suggested a misunderstanding of the randomisation process; for example, saying ‘… it (being in the trial) turned out not to even be worth it because we didn’t get the right envelope’.

Feedback from women who completed these two questionnaires is reassuring that the study was appropriately designed and relevant to those who participated. This is reflected in the higher than anticipated recruitment and good retention of participants, both of which are likely to be improved by well-designed studies integrated into existing health services that minimise inconvenience to participants [21, 22]. Nevertheless, women’s responses suggest issues for researchers to consider when planning future similar studies. For example, ways in which information about the trial might be made available earlier; providing additional information about the trial and/or the background condition that is available for those women who wish to access it; and providing more updates about the trial to participants.

A strength of this study is that we received responses from a high proportion of participants in the trial. Using three questions added to a follow-up questionnaire is simple, cost-effective, and time-efficient. Although the traditional method for assessing participants’ views is in-depth qualitative interviews, the responses to the questions used in our study are comparable with responses from studies using qualitative interviews [23]. Limitations of our study are that we do not know whether women who did not complete the questionnaire had a different experience of the Cord Pilot Trial. Also, women who were offered participation but declined or were not recruited for other reasons may have had different experiences and views. The experiences reported in this study may not be applicable to all parents who enrol their preterm baby into a clinical trial. Our results are based on a single trial, and other factors may be more or less important in trials with different risk and benefit profiles.

## Conclusions

Overall, women were positive about their experiences of participating in the Cord Pilot Trial, with only a small number negative about their participation. Women cited the importance of research, the benefits for their baby and benefits to others as reasons for participating. Women were positive about the level of information and explanations and the friendly and caring staff in the trial. Nevertheless, women had suggestions for how to improve the study. For example, some women would like to have received more information about the trial earlier in labour or during pregnancy; others would have liked more updates about progress. Some of the feedback also reflects misunderstanding about randomisation, a common finding in similar studies, suggesting that research is needed on how best to communicate this with potential trial participants. Responses also highlight the importance of communicating trial results to participants. This study demonstrates the value of simple, low-cost questionnaires to assess participants’ views of being in a randomised trial.

## Additional files

Additional file 1: Summary of free-text responses to explain response to ‘if time suddenly went backwards, and you had to do it all over again, would you agree to participate in the Cord Pilot Trial?’. (DOCX 38 kb)

Additional file 2: Summary of free-text responses to the two experience questions on the second questionnaire at 1 year. (DOCX 30 kb)
