# Supplementary material for: Women’s experiences of participating in a randomised trial comparing alternative policies for timing of cord clamping at very preterm birth: a questionnaire study
Source: Trials. 2019 Apr 16;20:225. doi: 10.1186/s13063-019-3325-4 (PMC6469101; doi:10.1186/s13063-019-3325-4)
Supplement: Supplementary file 1 — Summary of free-text responses to explain response to ‘if time suddenly went backwards, and you had to do it all over again, would you agree to participate in the Cord Pilot Trial?’. (DOCX 38 kb) [file 13063_2019_3325_MOESM1_ESM.docx]

**Summary of free text responses to explain response to “if time suddenly went backwards, and you had to do it all over again, would you agree to participate in the Cord pilot trial?”**

Table S1: For the first questionnaire, summary of free text responses to explain response to “if time suddenly went backwards, and you had to do it all over again, would you agree to participate in the Cord pilot trial?”

|  | **Consent pathway** | | **Allocated group** | | **Sample of comments** |
| --- | --- | --- | --- | --- | --- |
|  | **Usual one stage** | **Two stage** | **Clamping ≥2 mins** | **Clamping**  **≤20 secs** |  |
| ***‘definitely yes’*** | ***n=99*** | ***n=32*** | ***n=75*** | ***n=56*** |  |
| *Research is important* | 28 | 6 | 16 | 18 | “I feel it's important to contribute, where possible, to the evidence base for interventions and I had previously been aware of the findings for delayed cord clamping for term babies so can see that it may well have benefits for premature babies”  “I understand that research needs to be done in order to improve care further - I was happy to help with that”  “All these trials is the only way to learn about all future trials” |
| *Benefit to baby* | 21 | 5 | 20 | 6 | “I felt the cord delay clamping gave my baby a better chance due to him getting more of my blood and oxygen after birth”  “The trial gave my daughter more of a chance of surviving, and she thrived and is still continuing to do so. I'm really pleased I agreed to participate”  “I was randomised into the delay arm & I think the 2 min delay in cord clamping was very beneficial to my baby” |
| *Benefit to others* | 18 | 8 | 17 | 9 | “The care of my baby was great due to previous trials and questionnaires that have been done. This made me more than happy to help and be part of something that could help other prem babies and parents”  “I want to help other mums + babies in the same situation. In a small way I feel as though I am giving something back”  “If it helps babies in future why would we not do it. If it’s no harm to our baby and potentially helps others then everyone should do it” |
| *No risk to baby* | 15 | 7 | 9 | 13 | “As long as there is no harm to our babies we are always happy to help with future research to help future babies”  “I will participate because I see no harm on doing that, as long as it won’t harm my baby, there is no reason for not trying to do it again”  “The trial had no negative effect on the birth of my baby, and did not distress me in any way. I can see no negative aspect of participating in research that can benefit other premature babies in the future” |
| *Positive about trial aims* | 10 | 2 | 8 | 4 | “I think it's great what you’re doing, in a natural birth the idea that the baby is cared for you next to you on the bed, as it would be very comforting to me, as I has a section, under anaesthetic I didn't know anything different”  “It's important! - Also it made sense. If I gave birth alone, as many women across the world do, the cord would remain attached. No other mammals or beings use a knife or scissors to cut the cord immediately. It seems natural to keep the cord attached”  “The whole reason for doing it just made sense to me and made me wonder why delayed cord clamping is not routine anyway” |
| *Positive experience with trial* | 7 | 3 | 3 | 7 | “It was all very good. Simple to sign up lots of information given and all for a good trial that would be interesting to know the results. Already knew a bit about it before and wanted to try”  “Because it was all well explained & I could see what was going on” |
| *No inconvenience* | 3 | 2 | 4 | 1 | “The trial is not intrusive and does not require masses of direct participation”  “It has not been very intrusive” |
| ***‘probably yes’*** | ***n=28*** | ***n=8*** | ***n=17*** | ***n=19*** |  |
| *No risk to baby* | 5 | 2 | 4 | 3 | “Yes because I can’t see it doing any harm”  “It doesn’t harm your baby so I don’t see why not” |
| *Benefit to others* | 5 | 0 | 1 | 4 | “We were glad that participating may help other babies in the future”  “It's very helpful to other so I wouldn't mind helping ever understand what's what so they understand and get best knowledge to help them through this hard time” |
| *Research is important* | 2 | 1 | 1 | 2 | “If there wasn't any trials then we wouldn't see if things worked” |
| *Benefit to baby* | 1 | 2 | - | 3 | “Given my circumstances, I would probably say yes to anything that could help/benefit my baby (very premature)”  “Because this could be potentially good for my baby” |
| *Time and information* | 3 | - | 1 | 2 | “I would have liked more time to understand the trial however I understand I had an emergency section, so there was no time” |
| *Other themes* | 2 | 3 | 2 | 3 | “Depended on the situation at the time had baby of not made a sound can’t say I would of allowed the wait to do the trial”  “Not had to do much other than to fill out questionnaire”  “I don't think being part of the trial affected the care me and the baby received” |
| ***‘probably no’*** | **n=1** | **n=0** | **n=0** | **n=1** |  |
| *No impact* | 1 | - | - | 1 | “I didn't feel it had any benefit or loss” |
| ***‘definitely no’*** | ***n=2*** | ***n=0*** | ***n=1*** | ***n=1*** |  |
| *Strong preference to be in one arm of the trial* | 2 | - | 1 | 1 | “To be honest I thought it was a waste of time for me. We had an envelope waved in our face when I was in theatre ready for my C section & was told the cord wouldn't be clamped & cut after 2 minutes. This was disappointing”  “Due to the emergency c-section I would not have wanted to wait the 2 minutes to get the babies to ICU” |
| *Negative experience* | 1 | - | - | 1 | “I also felt a bit hounded with the trial people always turning up unannounced & usually at an inconvenient time such as when I was with a doctor or midwife or had received distressing news” |
| ***‘not sure’*** | ***n=6*** | ***n=3*** | ***n=3*** | ***n=6*** |  |
| *Strong preference to be in one arm of the trial* | - | 1 | 1 | - | “Knowing it most likely benefited my baby who received delayed cord clamping I am not sure I would participate in the trial since there was a chance I would not have had delayed clamping since the trial is randomised” |
| *Misunderstanding of trial* | 1 | - | - | 1 | “It's not much to do with the cord its more about the Resus trolley being next to you. I thought it was to do with the cord & they came to examine it or something” |
| *Circumstances at time of recruitment* | - | 1 | - | 1 | “At the time of randomisation, Dad says he had spent 24 hours in hospital, was tired and was not sure what he was consenting to. Did not really look at Patient Information leaflet properly after” |
| *Unable to participate in trial* | 1 | - | 1 | - | “We agreed, But due to the procedures during birth, it meant that it was not possible to continue during the operation” |

Table S2*:* For the second questionnaire, summary of free text responses to explain response to “if time suddenly went backwards, and you had to do it all over again, would you agree to participate in the Cord pilot trial?”

|  | **Consent pathway** | | **Allocated group** | | **Sample of comments** |
| --- | --- | --- | --- | --- | --- |
|  | **Usual one stage** | **Two stage** | **Clamping after ≥2 mins** | **Clamping**  **≤20 secs** |  |
| ***‘definitely yes’*** | ***n=81*** | ***n=22*** | ***n=69*** | ***n=34*** |  |
| *Benefit to others* | 28 | 7 | 19 | 16 | “If I can help others with this trial then I would gladly participate”  “We are happy to be part of research that will improve care” |
| *Research is important* | 18 | 5 | 16 | 7 | “I think things don't change unless trials are done, for things to progress and move on”  “The hospital has done so much for us, we feel that it is important to support any research to help babies and births there in the future”  “I believe it is important research and if it can benefit others in the future, it is worth participating in” |
| *Benefit to baby* | 17 | 5 | 20 | 2 | “I would agree because it helped my baby survive. It's a really good thing to do”  “Yes, I truly believe keeping the cord attached for those extra minutes helped him tremendously”  “I'm quite sure the extra minute the cord was attached was beneficial.” |
| *No risk to baby* | 6 | 4 | 7 | 3 | “I am more than happy to let my baby take part in any study as long as it doesn't affect him in any-way”  “As long as no harm comes to our babies we believe in helping with research if it helps other babies” |
| *Not impact on mother and/or birth* | 8 | 1 | 5 | 4 | “It didn't affect me in any way so there would be no reason not to partake”  “Did not impact birth experience” |
| *Positive about the trial aims* | 4 | - | 4 | - | “We believed in the trial 100% and had no concerns in participating. …so this was excellent as we believed in the benefits of the delay” |
| *Other* | 4 | 3 | 4 | 2 | “It's a very simple trial and didn't require us to do much”  “I was happy with the input” |
| ***‘probably yes’*** | ***n=8*** | ***n=11*** | ***n=11*** | ***n=8*** |  |
| *Benefit to others* | 2 | 4 | 1 | 5 | “Happy to be of help in the research if it might benefit babies in the future”  “I would like to participate in the Cord pilot trial because they put a lot of effort in research so we need to help them and hopefully in future their great effort will give a lot of benefits in health care and benefits many babies” |
| *Benefit to baby* | 3 | 1 | 3 | 1 | “If it would help my baby I would do anything”  “No regrets, I think it seemed to work out for the better” |
| *No risk to baby* | 3 | - | 2 | 1 | “I believe I would participate again as long as I was convinced it would not be detrimental to baby’s health” |
| *Uncertain of the impact of the trial* | 2 | 1 | 2 | 1 | “I don't mind participating as it would be helpful for premature babies but it is not very clear to us if it is beneficial to our son in any way...?” |
| *Simple trial* | - | 2 | - | 2 | “It was easy to be part of it” |
| *Other* |  |  | 2 | 1 | “I wasn't happy at the timing I was asked to do this. I was asked as I was about to push my baby out and didn't really understand what I was agreeing to” |
| ***‘probably no’*** | ***n = 2*** | ***n = 0*** | ***n=1*** | ***n=1*** |  |
| *Less of a priority when baby born* | 2 | - | 1 | 1 | “Although I feel this is great to find new ways of doing things, I was too busy thinking about my baby being premature + never fully took all the info in” |
| ***‘definitely no’*** | ***n=1*** | ***n=0*** | ***n=0*** | ***n=1^†^*** |  |
| ***‘not sure’*** | ***n=6*** | ***n=0*** | ***n=1*** | ***n=5*** |  |
| *Can’t see benefit* | 1 | - | - | 1 | “I can't see the benefit of this to [baby’s name]” |
| *Need to see results* | 1 | - | - | 1 | “Don't know results of the trial. Personally, wouldn't know if that makes a difference / % of results, to make an informed decision. Would feel more comfortable to make decision knowing results” |

***^†^*** only one respondent selected this and did not give an explanation
