# Supplementary material for: Women’s experiences of participating in a randomised trial comparing alternative policies for timing of cord clamping at very preterm birth: a questionnaire study
Source: Trials. 2019 Apr 16;20:225. doi: 10.1186/s13063-019-3325-4 (PMC6469101; doi:10.1186/s13063-019-3325-4)
Supplement: Supplementary file 2 — Summary of free-text responses to the two experience questions on the second questionnaire at 1 year. (DOCX 30 kb) [file 13063_2019_3325_MOESM2_ESM.docx]

**Summary of free text responses to the three experience questions on the second questionnaire at one year.**

**Table S1:** For the second questionnaire, summary of responses to “Please tell us if there was anything about the Cord pilot trial that you think could have been done better”

| *Theme* | **Consent pathway** | | **Allocated group** | | **Sample of comments** |
| --- | --- | --- | --- | --- | --- |
|  | **One stage**  **n = 64** | **Two stage**  **n = 21** | **Clamping ≥2 mins**  **n = 51** | **Clamping**  **≤20 secs**  **n = 34** |  |
| *Nothing could have been done better* | 44 | 17 | 37 | 24 | “No - think it's been perfect” |
| Approach earlier | 3 | 3 | 5 | 1 | “If I was asked to do it a bit earlier on during my labour then I may have understood it more” |
| More updates and information about trial | 3 | 2 | 2 | 3 | “I would like to have been informed of the future plans of the trial”  “I'd like more feedback on results however I presume it is yet to be concluded” |
| Better timed follow-up | 3 | - | 2 | 1 | “I felt a little harassed for forms while my baby lay in an incubator. A little longer wait for the first questionnaire would be great”  “Too much information to take in all at once. Sometimes I was with baby and there were letters and questionnaires waiting for me. It was a bit hectic” |
| Decide earlier about arm of trial | 2 | - | 1 | 1 | “Maybe decide on whether it would be a delayed clamping in advance so that you know. Everything was very hectic and I only vaguely remember others talking about it. It was after he was born that I knew as I was told to move back as short cord” |
| No randomisation | 3 | - | 1 | 1 | “I think rather than the cord pilot trial being a 'fate' trial regards to picking out an envelope parents should be given the choice to take part or not” |
| Other suggestions | 5 | 1 | 3 | 3 | “I wasn't aware initially that the Doctor's would choose a card dictating whether cord to be cut straight away or left. I thought all the participants would have the cord attached for the period of time after delivery This could be explained better”  “be prepared for a short cord for a prem birth” |

TableS 2: For the second questionnaire, summary of responses to “Please tell us if there was anything about the Cord pilot trial, or your experience of joining the trial that you think was particularly good”

| *Theme* | **Consent pathway** | | **Allocated group** | | **Sample of comments** |
| --- | --- | --- | --- | --- | --- |
|  | **One stage**  **n=58** | **Two stage**  **n=21** | **Clamping ≥2 mins**  **n = 53** | **Cord clamping**  **≤20 secs**  **n = 26** |  |
| Good information and explanation | 18 | 4 | 18 | 4 | “It was explained really well so I fully knew what I was getting myself into”  “Well, I was glad that everything about the trial was explained to me clearly so I didn't have any doubts”  “Follow up a few days later to ensure we understood was helpful” |
| Caring and friendly staff | 15 | 5 | 13 | 7 | “[name of staff member] In one word. He was the Cord Pilot representative in the hospital at the time, hopefully still is. He is a testament to caring professions. He was so kind and understanding. He took time to come see us regularly & even helped with issues aside to the pilot scheme. At a time of huge stress in our lives he was a welcome sight”.  “The staff that came round were all lovely”.  “Is just the encouragement from the team that kept me going” |
| Benefit to baby | 5 | - | 4 | 1 | “My little boy only had to have 1 blood transfusion during his stay which the doctors seemed surprised about, I like to think that the delayed cord clamping that helped him” |
| Personable trial | 5 | - | 4 | 1 | “Also, the 1st birthday card you sent made me cry with happiness”  “Very lovely and made it not an impersonal trial experience” |
| Learning new interesting things | 3 | 1 | 3 | 1 | “Learning new things, and what is happening out there”  “It was quite good and interesting” |
| Benefit to others | 2 | 1 | 2 | 1 | “Glad to be part of a much bigger picture that could help other mothers and their baby”  “Opportunity to help with research” |
| Unobtrusive | - | 2 | 1 | 1 | “Not been involved in anything like this before so nothing to compare against really. It was very unintrusive..... I wouldn't have liked it if too much involvement was required” |
| No pressure from staff | 2 | - | - | 2 | “We didn't feel pressured at all to take part” |
| Seeing baby for longer | 2 | - | 1 | 1 | “Although I never got to experience it, the idea of seeing your baby next to you whilst the doctors work on him / her whilst still attached was a lovely thought” |
| Other | 5 | 3 | 5 | 3 | “The trial gave me hope”  “The fact that it's random - you don't have any control over what you get” |
